# Supplementary material for: Palmitic Acid-Conjugated Radiopharmaceutical for Integrin αvβ3-Targeted Radionuclide Therapy
Source: Pharmaceutics. 2022 Jun 23;14(7):1327. doi: 10.3390/pharmaceutics14071327 (PMC9321335; doi:10.3390/pharmaceutics14071327)
Supplement: Supplementary file 1 [file pharmaceutics-14-01327-s001.zip › pharmaceutics-1703986-supplementary.pdf]

Supplemental Information for

Palmitic Acid-Conjugated Radiopharmaceutical for Integrin $\alpha_v\beta_3$-Targeted Radionuclide Therapy

Guangjie Yang [1], Hannan Gao [2], Chuangwei Luo [1], Xiaoyu Zhao [1], Qi Luo [3], Jiyun Shi [2,*] and Fan Wang [1,2,3,*]

[1] Medical Isotopes Research Center and Department of Radiation Medicine, State Key Laboratory of Natural and Biomimetic Drugs, School of Basic Medical Sciences, Peking University, Beijing 100191, China; docyang@bjmu.edu.cn (G.Y.); luocw@bjmu.edu.cn (C.L.); 2011110025@bjmu.edu.cn (X.Z.)

[2] Key Laboratory of Protein and Peptide Pharmaceuticals, CAS Center for Excellence in Biomacromolecules, Institute of Biophysics, Chinese Academy of Sciences, Beijing 100101, China; gaohan-nan@ibp.ac.cn

[3] Guangzhou Laboratory, Guangzhou 510005, China; luo_qi@gzlab.ac.cn

[*] Correspondence: shijiyun@ibp.ac.cn (J.S.); wangfan@bjmu.edu.cn (F.W.)

This file includes

Method and results of Western Blotting.
Figure S1. The synthesis route of DOTA-Palm-3PRGD$_2$.
Figure S2. The synthesis route of DOTA-3PRGD$_2$.
Figure S3. The synthesis route of DOTA-Palm.
Figure S4. HPLC chromatogram results during the synthesis of DOTA-Palm-3PRGD$_2$.
Figure S5. HPLC chromatogram results during the synthesis of DOTA-Palm and DOTA-3PRGD$_2$.
Figure S6. MS analysis of DOTA-Palm-3PRGD$_2$.
Figure S7. MS analysis of DOTA-3PRGD$_2$.
Figure S8. MS analysis of DOTA-Palm.
Figure S9. Radio-HPLC chromatogram results of (A) ^{177}Lu-Palm-3PRGD$_2$, (B) ^{177}Lu-Palm and (C) ^{177}Lu-3PRGD$_2$. (D) In vitro stability analysis of ^{177}Lu-Palm-3PRGD$_2$.
Figure S10. Blood clearance curves of ^{177}Lu-Palm-3PRGD$_2$ and ^{177}Lu-3PRGD$_2$ performed in C57BL/6 mice.
Figure S11. H&E staining of major organs after treatment study.
Figure S12. Integrin α_v, β_3 and β_5 expression patterns in U87MG and MC38 cell lines and tumor tissues.
Table S1. Biodistribution results of ^{177}Lu-Palm-3PRGD$_2$ in MC38-tumor bearing mice.
Table S2. Comparison of biodistribution results of ^{177}Lu-Palm-3PRGD$_2$, ^{177}Lu-3PRGD$_2$, and ^{177}Lu-Palm at 4 h p.i. in MC38-tumor bearing mice.
Table S3. Comparison of biodistribution results of ^{177}Lu-Palm-3PRGD$_2$, ^{177}Lu-3PRGD$_2$, and ^{177}Lu-Palm at 12 h p.i. in MC38-tumor bearing mice.
Table S4. Tumor-to-tissue ratios of ^{177}Lu-Palm-3PRGD$_2$.
Table S5. Comparison of tumor-to-tissue ratios of radiopharmaceuticals at 4 h and 12 h p.i.
Table S6. Human absorbed effective dose estimates of ^{177}Lu-Palm-3PRGD$_2$ obtained from MC38

tumor mice (mSv/MBq, n = 4).

Western Blotting

The U87MG tumor-bearing BALB/c nude mice and MC38 tumor-bearing C57BL/6 mice were euthanized and the tumor tissues were harvested. After homogenization, tumor tissue proteins were extracted. Besides, whole proteins of U87MG cells and MC38 cells were also extracted. The protein concentrations were determined by a bicinchoninic acid protein assay kit (Thermo Fisher Scientific, Waltham, MA). After sodium dodecyl sulfate-polyacrylamide gel electrophoresis (SDS-PAGE), the proteins were transferred onto a polyvinylidene difluoride membrane (Invitrogen) and blocked in 5% skimmed milk at room temperature for 1 h. Then the proteins were immunoblotted overnight at 4 °C with primary antibodies against integrin αv (1:1000; Abcam, ab179475), integrin β3 (1:1000; Cell Signaling Technolog, D7X3P) or integrin β5 (1:1000; Immunoway, YT2373). On the following day, membranes were rinsed and incubated with horseradish peroxidase-conjugated secondary antibodies (1:10000; Beyotime Biotechnology) for 1 h at room temperature. Bands were visualized after the addition of SuperSignal West Pico Plus Chemiluminescent Substrate (Thermo Fisher Scientific) and imaged with the Molecular Imager PharosFX Plus System (Bio-Rad Laboratories, Hercules, CA).

As shown in Figure S12, U87MG cells expressed integrins $\alpha_v\beta_3$ and $\alpha_v\beta_5$. MC38 cells showed low expression of integrin $\alpha_v\beta_3$, but no expression of integrin $\alpha_v\beta_5$. At the tissue level, U87MG tumors also expressed integrin $\alpha_v\beta_3$ and $\alpha_v\beta_5$, and the expression level of integrin $\alpha_v\beta_3$ was much higher than that of integrin $\alpha_v\beta_3$. In accordance with MC38 cells, MC38 tumors also expressed integrin $\alpha_v\beta_3$, but not integrin $\alpha_v\beta_5$.

Figure S1. The synthesis route of DOTA-Palm-3PRGD2.

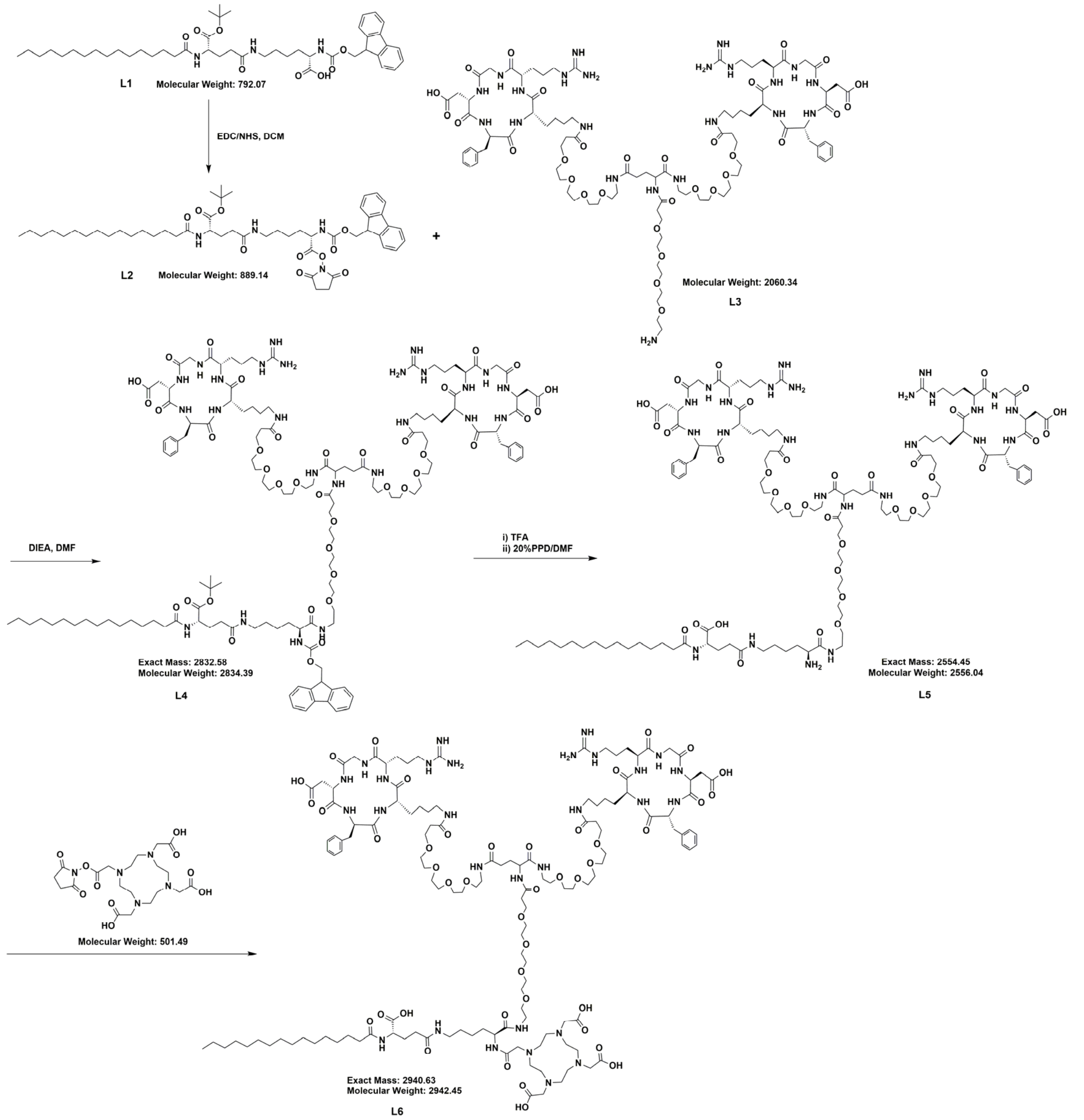

Figure S2. The synthesis route of DOTA-3PRGD2.

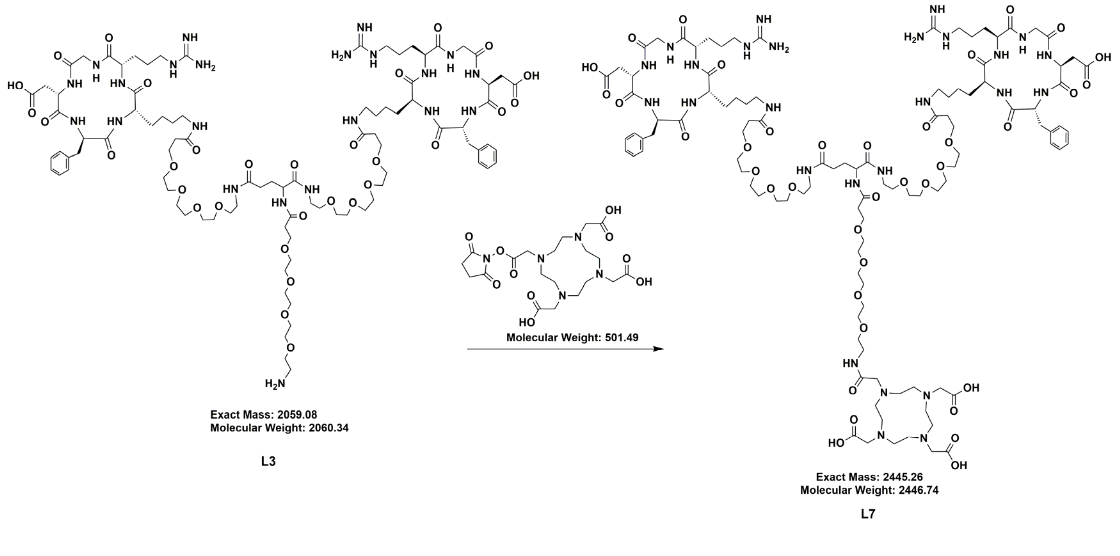

Figure S3. The synthesis route of DOTA-Palm.

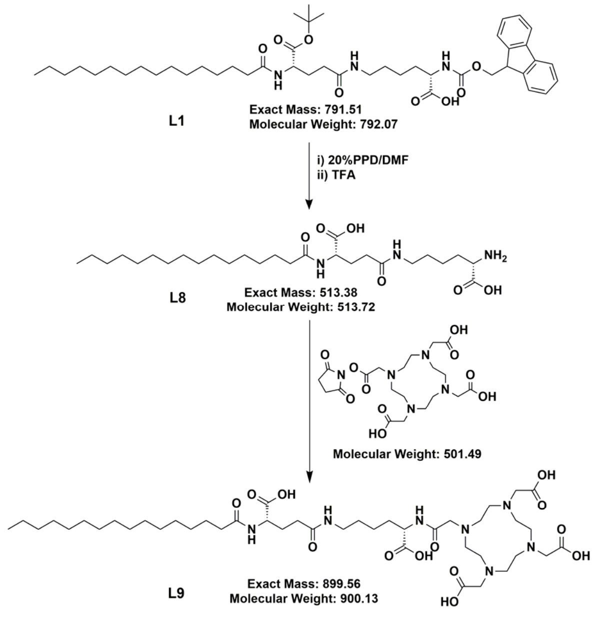

Figure S4. HPLC chromatogram results during the synthesis of DOTA-Palm-3PRGD2.

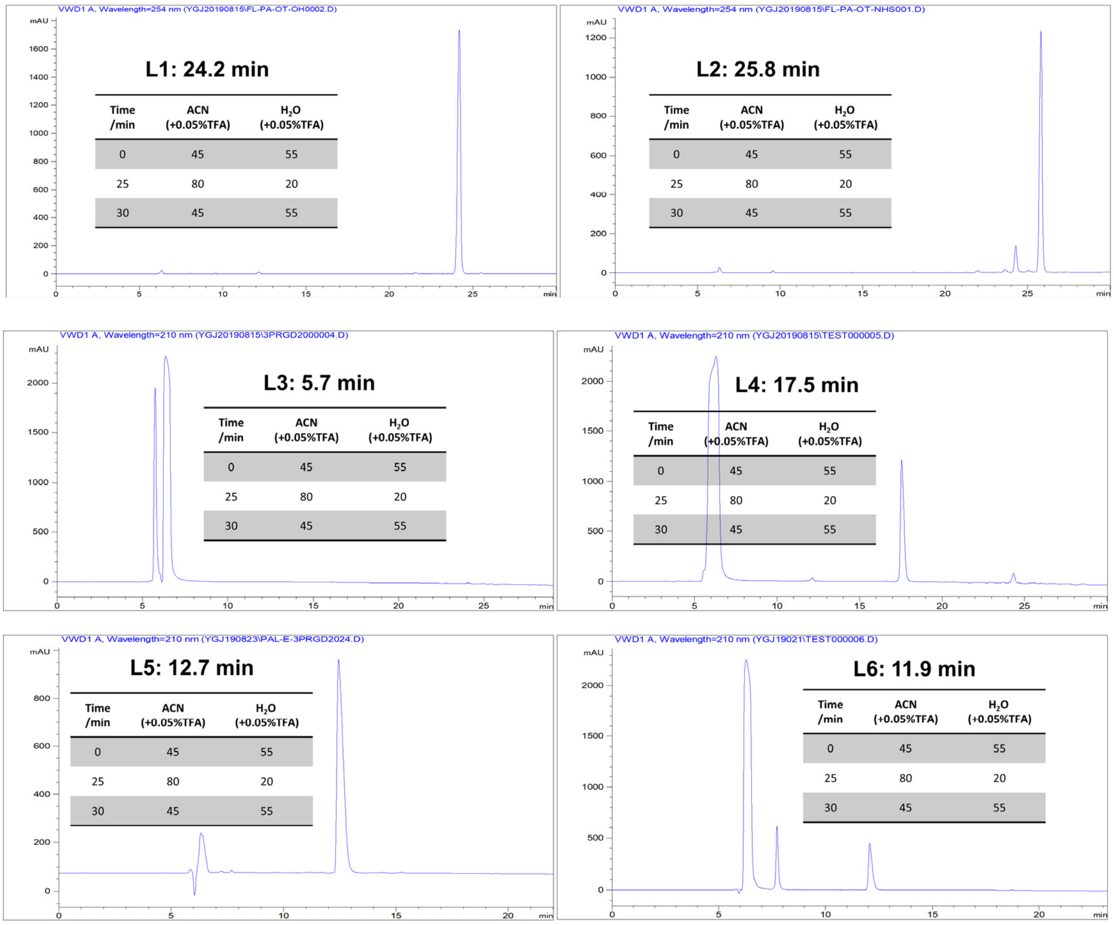

Figure S5. HPLC chromatogram results during the synthesis of DOTA-Palm and DOTA-3PRGD2.

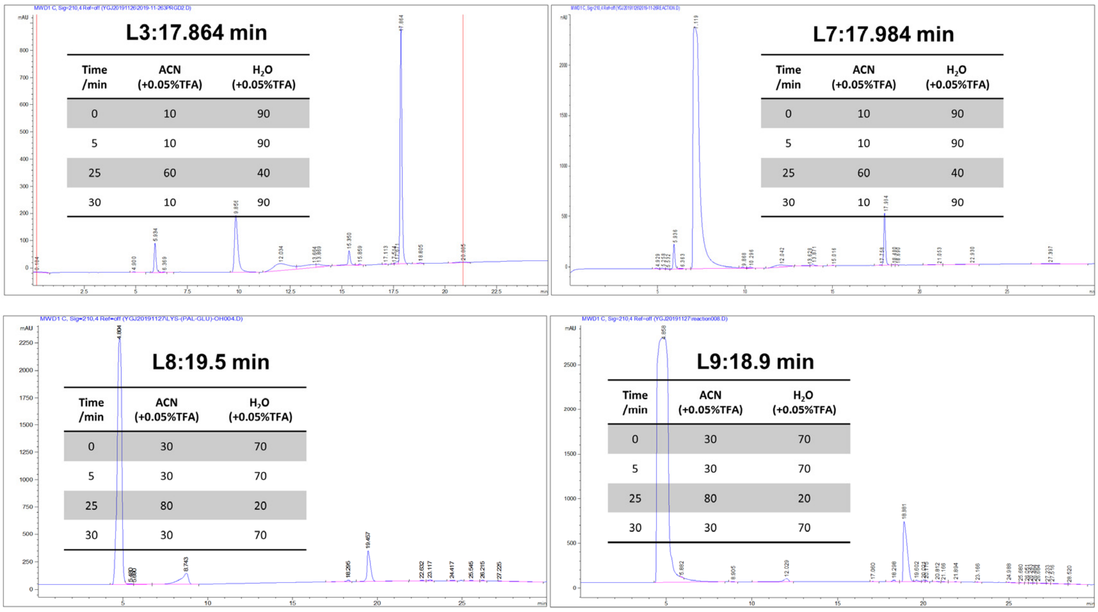

Figure S6. MS analysis of DOTA-Palm-3PRGD2.

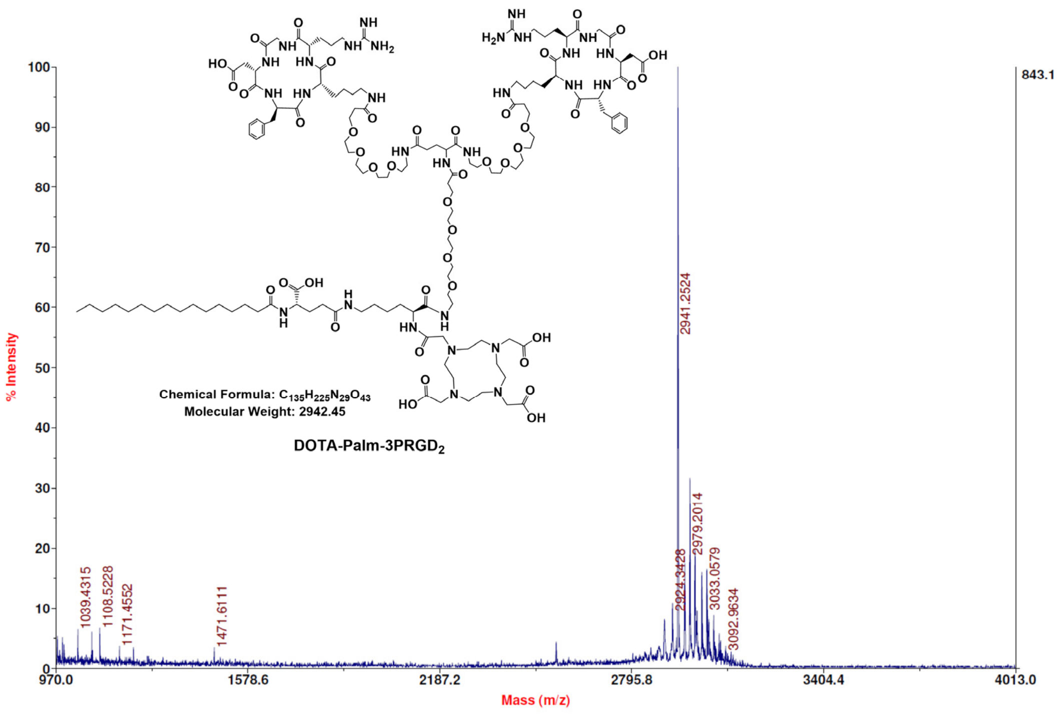

Figure S7. MS analysis of DOTA-3PRGD2.

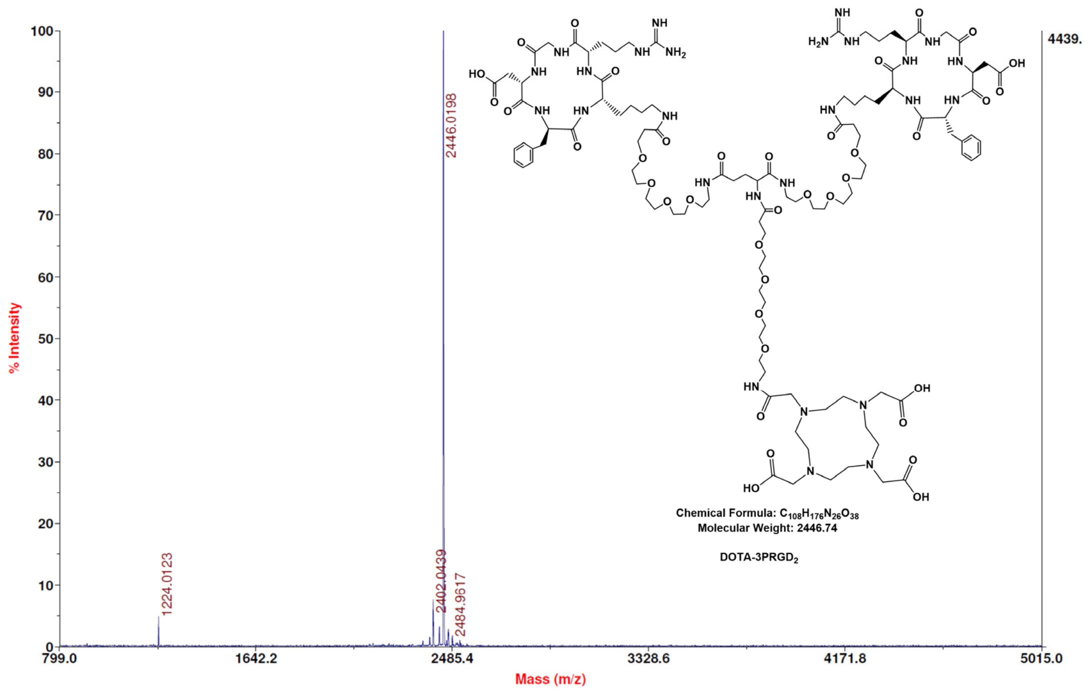

Figure S8. MS analysis of DOTA-Palm.

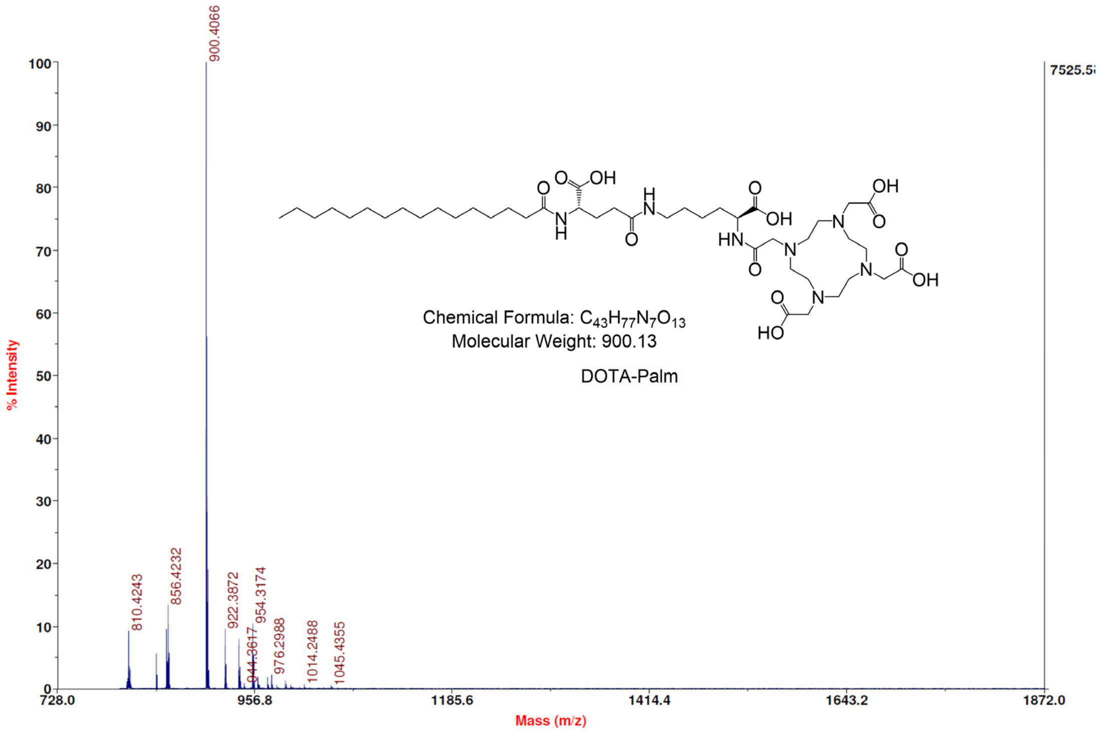

Figure S9. Radio-HPLC chromatogram of (A) ^{177}Lu-Palm-3PRGD$_2$, (B) ^{177}Lu-Palm and (C) ^{177}Lu-3PRGD$_2$. (D) In vitro stability analysis of ^{177}Lu-Palm-3PRGD$_2$. Stability of ^{177}Lu-Palm-3PRGD$_2$ in human serum was monitored from 1 h to 24 h using radio-HPLC.

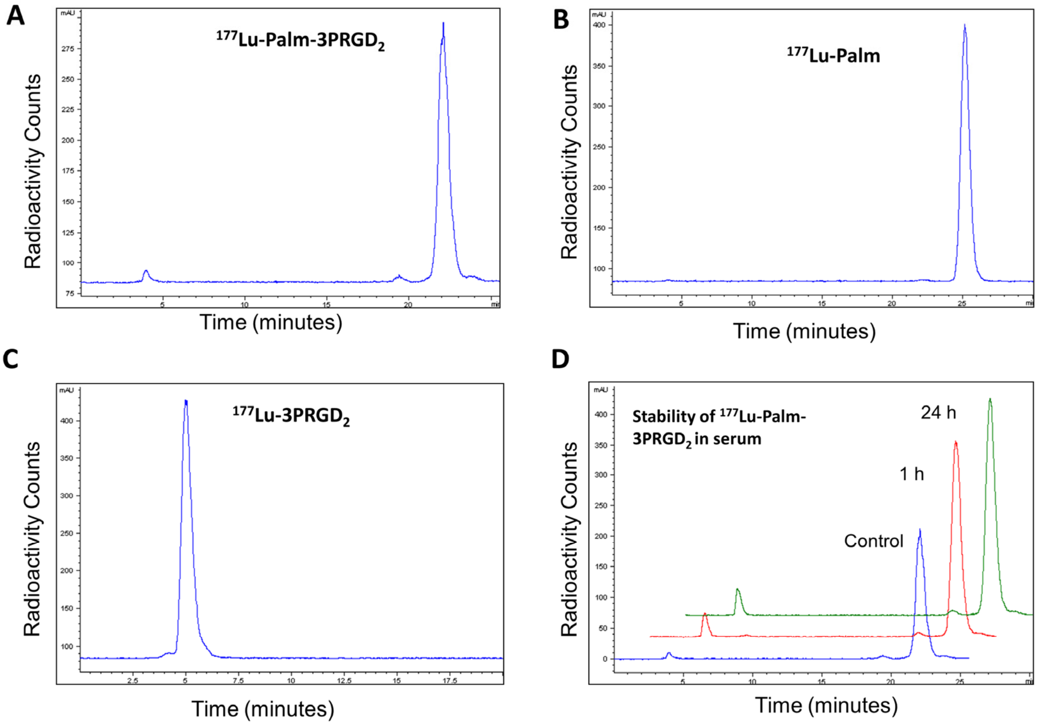

Figure S10. Blood clearance curves of ^{177}Lu-Palm-3PRGD$_2$ and ^{177}Lu-3PRGD$_2$ performed in C57BL/6 mice.

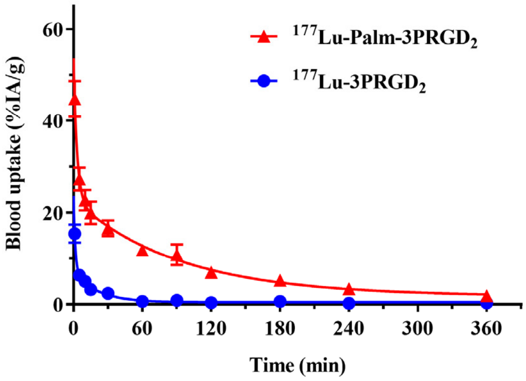

C57BL/6 mice (female, 20 g)	^{177}Lu-Palm-3PRGD$_2$	^{177}Lu-3PRGD$_2$
**Half Life (Slow)/min**	63.71	14.54
**Half Life (Fast)/min**	2.08	1.06

Figure S11. H&E staining of major organs including heart, liver, spleen, lung and kidney after treatment with ^{177}Lu-Palm-3PRGD$_2$, ^{177}Lu- 3PRGD$_2$, ^{177}Lu-Palm, and PBS, respectively.

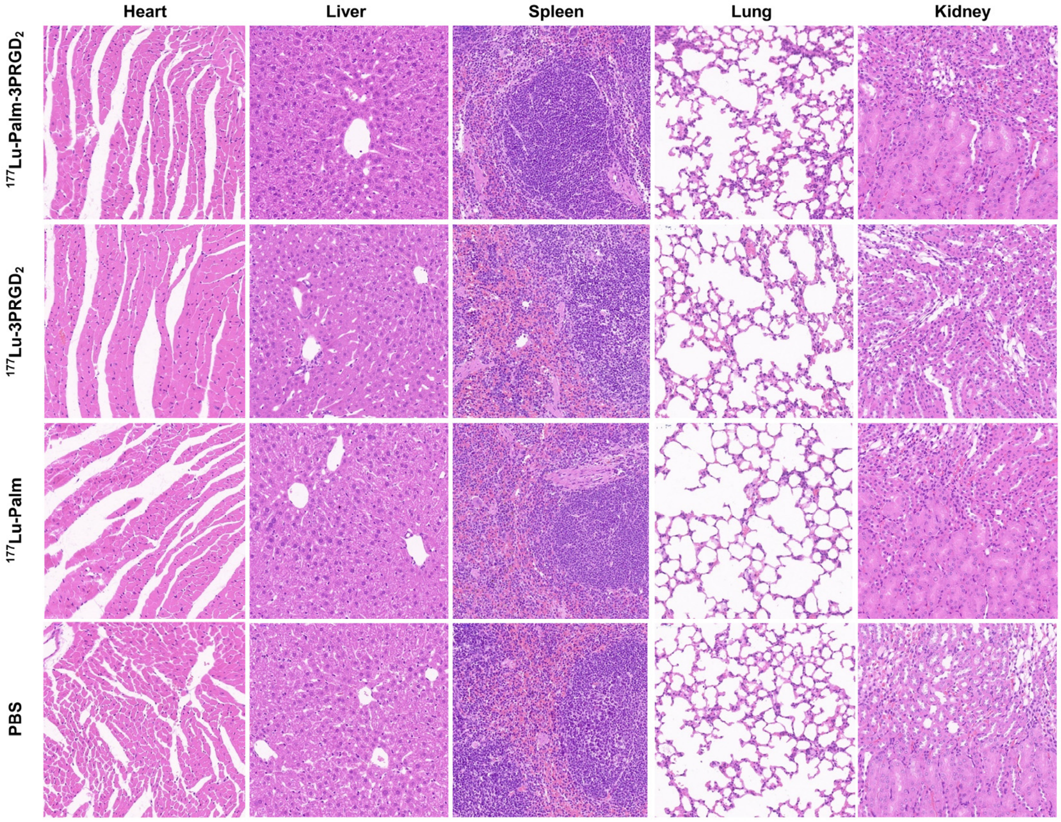

Figure S12. Integrin α_v, β_3 and β_5 expression patterns in U87MG and MC38 cell lines and tumor tissues.

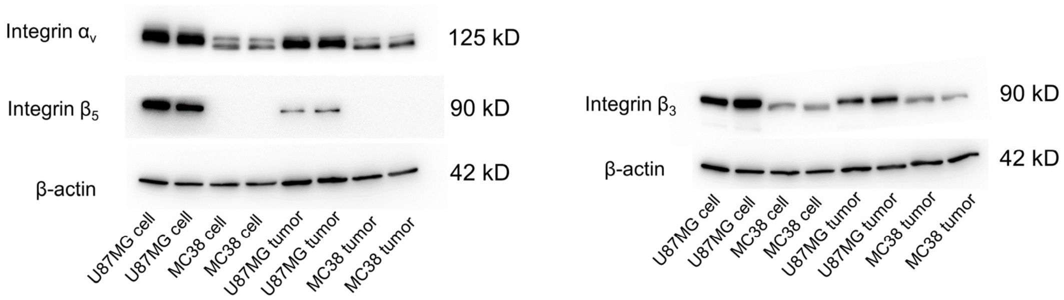

Table S1. Biodistribution results of ^{177}Lu-Palm-3PRGD$_2$ in MC38 tumor bearing mice.

Biodistribution results of ^{177}Lu-Palm-3PRGD$_2$ (%ID/g, n = 4)

Groups / Organs	1 h		1h(Block)		4 h		12 h		24 h		72 h	
	Mean	SD	Mean	SD	Mean	SD	Mean	SD	Mean	SD	Mean	SD
**Blood**	9.73	1.16	27.88	4.66	4.11	1.52	0.58	0.22	0.24	0.03	0.21	0.03
**Liver**	13.70	2.87	5.97	1.66	22.40	2.55	22.05	1.59	13.69	1.94	6.73	1.18
**Spleen**	7.35	2.02	4.53	1.42	12.41	2.09	18.39	1.16	14.94	1.29	8.38	1.42
**Stomach**	5.73	1.53	4.32	0.88	7.22	2.39	6.07	0.17	3.70	0.64	1.38	0.23
**Intestine**	20.65	1.97	7.53	2.15	17.63	0.87	21.15	1.00	17.37	2.97	12.80	1.06
**Kidney**	17.65	3.05	10.48	2.88	20.11	0.60	19.31	0.14	17.04	1.44	10.58	1.11
**Heart**	5.59	1.02	5.63	1.77	4.93	1.00	3.20	0.01	2.70	0.20	1.53	0.09
**Lung**	7.79	1.65	10.36	2.89	7.85	2.08	5.20	0.13	3.53	0.39	1.55	0.24
**Bone**	3.39	0.76	2.78	0.63	4.55	1.80	3.05	0.10	3.13	0.54	1.97	0.22
**Muscle**	1.96	0.20	2.20	0.91	1.70	0.46	1.24	0.07	0.96	0.05	0.70	0.07
**Tumor**	14.41	2.53	7.00	2.47	26.27	6.34	22.91	4.20	17.22	3.30	5.83	1.27
**Brain**	0.52	0.05	0.49	0.18	0.55	0.15	0.43	0.04	0.40	0.07	0.29	0.01

Table S2. Comparison of biodistribution results of ^{177}Lu-Palm-3PRGD$_2$, ^{177}Lu-3PRGD$_2$ and ^{177}Lu-Palm at 4 h p.i. in MC38 tumor bearing mice.

Biodistribution results of tracers at 4 h p.i. (%ID/g, n = 4)						
Tracers / Organs	**^{177}Lu-Palm-3PRGD$_2$**		**^{177}Lu-Palm**		**^{177}Lu-3PRGD$_2$**	
	Mean	SD	Mean	SD	Mean	SD
**Blood**	4.11	1.52	0.42	0.03	0.42	0.26
**Liver**	22.40	2.55	11.00	0.90	2.95	0.17
**Spleen**	12.41	2.09	0.44	0.05	2.65	0.23
**Stomach**	7.22	2.39	0.51	0.05	2.96	0.19
**Intestine**	17.63	0.87	1.26	0.47	9.85	1.83
**Kidney**	20.11	0.60	0.81	0.07	4.91	0.51
**Heart**	4.93	1.00	0.21	0.02	0.75	0.05
**Lung**	7.85	2.08	0.54	0.06	1.73	0.23
**Bone**	4.55	1.80	0.51	0.11	1.41	0.10
**Muscle**	1.70	0.46	0.13	0.01	0.52	0.09
**Tumor**	26.27	6.34	0.80	0.09	6.22	0.62
**Brain**	0.55	0.15	0.06	0.01	0.09	0.02

Table S3. Comparison of biodistribution results of ^{177}Lu-Palm-3PRGD$_2$, ^{177}Lu-3PRGD$_2$ and ^{177}Lu-Palm at 12 h p.i. in MC38 tumor bearing mice.

**Biodistribution results of tracers at 12 h p.i. (%ID/g, n = 4)**						
**Tracers / Organs**	**^{177}Lu-Palm-3PRGD$_2$**		**^{177}Lu-Palm**		**^{177}Lu-3PRGD$_2$**	
	Mean	SD	Mean	SD	Mean	SD
**Blood**	0.58	0.22	0.22	0.00	0.32	0.13
**Liver**	22.05	1.59	7.40	1.15	1.93	0.17
**Spleen**	18.39	1.16	0.43	0.01	1.89	0.31
**Stomach**	6.07	0.17	0.44	0.03	2.39	0.10
**Intestine**	21.15	1.00	0.77	0.27	7.97	0.53
**Kidney**	19.31	0.14	0.59	0.08	4.48	0.46
**Heart**	3.20	0.01	0.15	0.03	0.63	0.06
**Lung**	5.20	0.13	0.35	0.02	1.46	0.29
**Bone**	3.05	0.10	0.61	0.02	1.46	0.18
**Muscle**	1.24	0.07	0.16	0.01	0.64	0.10
**Tumor**	22.91	4.20	0.68	0.05	4.11	0.70
**Brain**	0.43	0.04	0.06	0.03	0.11	0.03

Table S4. Tumor-to-tissue ratios of ^{177}Lu-Palm-3PRGD$_2$.

Tumor-to-tissue ratios of ^{177}Lu-Palm-3PRGD$_2$ (n = 4)												
Groups / Tissues	1 h		1 h (Block)		4 h		12 h		24 h		72 h	
	Mean	SD	Mean	SD	Mean	SD	Mean	SD	Mean	SD	Mean	SD
**Blood**	1.47	0.09	0.25	0.08	7.28	1.53	44.87	14.78	73.21	12.00	28.17	5.25
**Liver**	1.06	0.04	1.19	0.38	1.02	0.16	1.05	0.12	1.30	0.46	0.89	0.31
**Spleen**	2.02	0.35	1.61	0.59	2.56	0.92	1.40	0.34	1.35	0.36	0.70	0.07
**Stomach**	2.57	0.32	1.64	0.53	4.06	0.78	3.84	0.63	4.74	1.03	4.25	0.79
**Intestine**	0.77	0.01	0.96	0.34	1.62	0.59	1.06	0.13	1.00	0.14	0.45	0.08
**Kidney**	0.82	0.05	0.69	0.24	1.42	0.50	1.20	0.18	1.01	0.17	0.56	0.17
**Heart**	2.59	0.24	1.32	0.55	6.12	1.65	7.11	1.09	6.41	1.45	3.86	1.07
**Lung**	1.87	0.16	0.71	0.27	3.98	1.24	3.86	1.36	4.89	0.84	3.78	0.60
**Bone**	4.30	0.34	2.51	0.69	6.66	1.55	7.14	1.57	5.61	1.25	2.96	0.57
**Muscle**	7.33	0.64	3.53	1.75	17.49	3.89	17.96	4.05	17.89	3.12	8.33	1.16
**Brain**	27.85	2.22	14.82	5.04	55.26	15.11	52.51	5.52	42.49	2.55	19.87	3.85

Table S5. Comparison of tumor-to-tissue ratios of radiopharmaceuticals at 4 h and 12 h p.i..

**Tumor-to-tissue ratios of radiopharmaceuticals (n = 4)**												
	**^{177}Lu-Palm-3PRGD$_2$**				**^{177}Lu-3PRGD$_2$**				**^{177}Lu-Palm**			
**Groups**	4 h		12 h		4 h		12 h		4 h		12 h	
**Tissues**	Mean	SD	Mean	SD	Mean	SD	Mean	SD	Mean	SD	Mean	SD
**Blood**	7.28	1.53	44.87	14.78	20.30	12.03	14.25	4.54	1.89	0.21	3.11	0.21
**Liver**	1.02	0.16	1.05	0.12	2.11	0.11	2.13	0.38	0.07	0.01	0.10	0.01
**Spleen**	2.56	0.92	1.40	0.34	2.36	0.36	2.22	0.57	1.82	0.04	1.59	0.16
**Stomach**	4.06	0.78	3.84	0.63	2.10	0.10	1.72	0.25	1.57	0.29	1.55	0.21
**Intestine**	1.62	0.59	1.06	0.13	0.66	0.21	0.52	0.11	0.68	0.19	0.96	0.40
**Kidney**	1.42	0.50	1.20	0.18	1.27	0.08	0.92	0.19	0.98	0.06	1.17	0.07
**Heart**	6.12	1.65	7.11	1.09	8.31	0.83	6.53	0.67	3.81	0.62	4.54	0.43
**Lung**	3.98	1.24	3.86	1.36	3.60	0.17	2.84	0.49	1.20	0.70	1.98	0.26
**Bone**	6.66	1.55	7.14	1.57	4.41	0.32	2.82	0.50	1.63	0.40	1.12	0.04
**Muscle**	17.49	3.89	17.96	4.05	12.00	1.36	6.60	2.01	5.96	0.44	4.19	0.05
**Brain**	55.26	15.11	52.51	5.52	68.65	17.96	39.94	13.67	14.69	3.90	14.69	9.18

Table S6. Human absorbed effective dose estimates of ^{177}Lu-Palm-3PRGD$_2$ obtained from MC38 tumor mice (mSv/MBq, n = 4).

Target Organ	Effective Dose (mSv/MBq)
Adrenals	1.40×10^{-5}
Brain	2.77×10^{-5}
Breasts	5.88×10^{-5}
Gallbladder Wall	0.00
LLI Wall	4.38×10^{-4}
Small Intestine	9.56×10^{-4}
Stomach Wall	2.73×10^{-3}
ULI Wall	1.91×10^{-5}
Heart Wall	0.00
Kidneys	9.93×10^{-4}
Liver	1.39×10^{-2}
Lungs	8.97×10^{-3}
Muscle	7.77×10^{-5}
Ovaries	1.02×10^{-3}
Pancreas	1.53×10^{-5}
Red Marrow	2.96×10^{-4}
Osteogenic Cells	3.27×10^{-5}
Skin	1.03×10^{-5}
Spleen	1.06×10^{-2}
Testes	0.00
Thymus	4.55×10^{-6}
Thyroid	6.43×10^{-5}
Urinary Bladder Wall	1.11×10^{-4}
Uterus	1.19×10^{-5}
Total Body	4.04×10^{-2}
